# The impact of sports participation on the social integration of China's migrant population: a serial mediation model of life satisfaction and subjective well-being

**DOI:** 10.3389/fspor.2026.1825077

**Published:** 2026-06-24

**Authors:** Xiaochu Yang, Xiang Fu, Linying Xiao, Zexin Wu

**Affiliations:** 1 Guangdong Polytechnic Normal University, Guangzhou, China; 2 Hunan University of Technology, Zhuzhou, China

**Keywords:** life satisfaction, migrant populations, social integration, sports participation, subjective well-being

## Abstract

**Background:**

Large-scale internal migration has become an important feature of China's urbanization, yet internal migrants often face challenges in social connection, community participation, and sense of belonging in destination cities. Although previous studies have linked sport participation with health, well-being, and social connection, less is known about how different forms of sport participation influence the social integration of internal migrants through psychological mechanisms.

**Objective:**

This study examined the influence of sports participation on the social integration of China's migrant population, focusing on the mediating roles of subjective well-being and life satisfaction, along with age-related differences.

**Methods:**

A sample of 1,660 migrants from the CGSS 2023 dataset was analyzed using Pearson correlation analysis and the PROCESS macro (Model 6) for SPSS to evaluate the serial mediation model. Additionally, heterogeneity analysis was performed across different age groups.

**Results:**

(a) Both physical exercise and sports spectatorship significantly and positively predicted social integration; (b) physical exercise enhanced social integration through the mediating effects of subjective well-being and life satisfaction; (c) sports spectatorship did not significantly predict subjective well-being or life satisfaction, and its indirect effect lacked consistent statistical support, indicating that the effect was primarily direct; (d) age-group analysis demonstrated that subjective well-being maintained relatively stable explanatory power in the relationship between physical exercise and social integration, while the indirect effect of spectatorship was statistically supported only in the older cohort.

**Conclusion:**

Participation in sports plays a significant role in promoting the social integration of migrant populations. However, the psychological mechanisms associated with various forms of participation exhibit distinct characteristics: engagement through physical exercise tends to be more stable, whereas the impact of spectatorship is influenced by age and specific contextual factors.

## 1 Introduction

### 1.1 Migration and social integration

Economic globalization and urbanization have accelerated migration toward urban centers that provide greater opportunities (1, 2). This trend is supported by advancements in transportation and information technology. Individuals relocate for various reasons, including education, employment, marriage, and family, necessitating adaptation to new environments (3, 4). However, migrants often encounter challenges in forming social connections and establishing stable routines, frequently facing limited integration with local communities (5, 6). Restricted interactions with residents can hinder feelings of belonging and identity, leading to weaker community ties and heightened social marginalization. This marginalization can diminish social trust and cohesion, thereby presenting challenges for governance (7).

### 1.2 Sports participation as a pathway to social integration

Sports participation, as an accessible, sustainable, and inclusive daily practice, provides migrants with opportunities for interaction and social connection that transcend identity boundaries (8). Research indicates that engagement in sports fosters frequent contact and cooperative interaction, which helps expand social networks, build social support, and enhance interpersonal trust, thereby offering a behavioral pathway to social integration (5, 7). However, the relationship between sports participation and integration is not merely linear; the critical factor is whether such participation leads to positive psychological adaptation and increased motivation for social engagement. For example, participation may enhance subjective well-being by improving positive emotional experiences, which subsequently influences migrants' cognitive evaluation of their quality of life, specifically, life satisfaction (9). When both subjective well-being and life satisfaction are elevated, migrants are more likely to adopt open attitudes toward social interaction and demonstrate a willingness to engage in public life, thus facilitating integration. Therefore, subjective well-being and life satisfaction may be essential for elucidating the psychological mechanisms through which sports participation influences social integration.

### 1.3 Sport, migration, and China's internal migrants

In research on sport and migrant social integration, studies conducted in different contexts have examined how sport participation helps migrants form social ties and engage with local community life. Drawing on recreational sport participation among Somali Australians, Spaaij (8) showed that sport can help migrants extend social relationships beyond existing ethnic, friendship-based, and kinship networks. Adler Zwahlen et al. (10) and Buser et al. (11), focusing on young immigrants and people with a migration background in Swiss sport clubs, suggested that sport clubs are not only spaces for physical activity but may also serve as organizational settings for developing social relationships and a sense of belonging. Related research on immigrant sport participation has also linked sport-based interaction with sense of community and subjective well-being (12).These studies also indicate that the integrative effects of sport participation are shaped by interaction opportunities, organizational openness, and the social climate of clubs. Taken together, they provide useful reference points for understanding the relationship between sport participation and migrant integration. However, existing research has mainly focused on cross-border immigrants, sport clubs, or specific community sport settings, while less attention has been paid to how sport participation influences social integration in the context of large-scale internal migration within a single country. Moreover, relatively few studies have examined different forms of everyday sport participation, psychological experiences, and social integration within a unified analytical framework.

In China, cross-regional population movement has emerged as a defining characteristic of socioeconomic development (13). According to data from the Seventh National Population Census, the migrant population reached 376 million in 2020, reflecting an increase of nearly 70% since 2010 (14). This demographic plays a vital role in urban labor supply and local economic development, with its size and flow patterns closely associated with urban vitality and regional progress (2, 15). For example, in Zhejiang Province, each additional 10,000 migrants contributes approximately RMB 1.182 billion to the provincial GDP (16). Despite these economic contributions, migrants continue to experience disparities compared to local residents in income, quality of life, and sense of belonging. In light of the increasing levels of sports participation in recent years, sports, as accessible and interactive public activities, may provide a valuable opportunity for China's internal migrants to expand their social networks, enhance their sense of belonging, and facilitate social integration. Thus, this study employs data from the 2023 China General Social Survey to examine the impact of sports participation on the social integration of the migrant population and to investigate the underlying mechanisms. The objective is to present a novel perspective on migrant integration in China and to provide empirical evidence for more inclusive community governance, while offering a comparative reference for future studies on domestic migration, local mobility, and community-based sport integration in other societies.

## 2 Literature review and research hypotheses

### 2.1 Defining social integration

The concept of social integration emerged from sociological investigations into the connections between individuals and their communities, highlighting the strength of individual-society bonds and the extent of community cohesion (17). Early studies often framed social integration as an individual's adaptation to social norms and communal values, emphasizing the integration of social relationships and the preservation of social order. However, moving beyond this rule-based adaptation perspective, Tajfel et al. (18) introduced a social-psychological dimension, positing that social integration encompasses not only behavioral participation in social networks but also the psychological development of a subjective sense of group belonging and identity.

As research has increasingly focused on immigrant and cross-regional migrant populations, the conceptualization of social integration has shifted from a unidimensional adaptation perspective to a multidimensional framework. A notable example is Entzinger and Biezeveld's (63) four-dimensional model, which includes economic, political, cultural, and social acceptance. This framework not only addresses the socio-ecological adaptation of migrants during intergroup interactions but also emphasizes internal processes such as psychological changes, behavioral adjustments, and stress responses associated with adaptation (69). In summary, this study defines social integration as the process by which migrants establish a foundation for livelihood in their host society, cultivate social connections, engage in public life, and achieve social acceptance.

Previous research on the determinants of social integration has predominantly concentrated on institutional and resource conditions, highlighting external factors such as household registration (hukou), employment, income, and access to public services (19–22). Nevertheless, it is important to recognize that social integration is not exclusively influenced by these external conditions. Throughout the migration and adaptation process, migrants’ psychological states and cognitive evaluations significantly affect their willingness to engage socially, their interaction patterns, and their overall assessment of urban life. These internal factors are essential in the transition from “objective embeddedness” to “subjective identification” (4, 23). In contrast to structural conditions, these psychological and cognitive factors are more amenable to change in daily life. However, the specific behaviors through which migrants can enhance their psychological states to promote integration remain insufficiently explored.

Sports participation, as a daily activity that integrates emotional regulation with social interaction, provides migrants with accessible opportunities for social engagement. This involvement allows them to access public spaces and social networks, whether through active participation or as spectators (24, 25). Consequently, it is essential to investigate how sports participation affects the social integration of migrant populations and to compare the varying impacts and underlying mechanisms associated with different forms of participation, including physical exercise and sports spectatorship. Such an investigation can establish a foundation for targeted interventions designed to enhance social integration through sports.

### 2.2 Hypothesis 1

Sports participation is widely acknowledged as an essential social activity that facilitates individuals’ entry into social networks and the formation of group bonds (26). In everyday contexts, sports activities serve as an accessible platform for interaction, enabling individuals from varied backgrounds to collaborate and communicate. This interaction fosters social connections and enhances group belonging and social identity (27). Within social environments such as schools and communities, sustained involvement in sports expands social circles, strengthens social support, and promotes adaptation and integration into the community. For example, sports activities can enhance individuals’ acceptance of communal life and their willingness to engage by improving peer interaction and group involvement (28, 29).

Sports participation includes not only physical exercise but also indirect forms such as sports spectatorship. Research indicates that viewing sports can enhance social bonds through shared emotional experiences and group identity, allowing individuals to develop a stronger sense of belonging and motivation for interaction (30, 31). Building on this research, the present study posits that both physical exercise and sports spectatorship may serve as significant avenues for fostering individual social interaction and group integration. Therefore, the following hypothesis is proposed:

**H1:** Sports participation (both physical exercise and sports spectatorship) has a significant positive predictive effect on social integration.

### 2.3 Hypothesis 2

Regarding the mechanisms through which sports participation affects the social integration of migrant populations, health status has been considered an important mediating factor. Regular physical exercise can alleviate psychological distress such as anxiety and depression, reduce stress load, and enhance individuals’ physical and mental resilience and life stability, thereby providing them with sufficient psychological and behavioral resources for social interaction and community participation in their destination cities (32, 33). Additionally, socioeconomic conditions are thought to constitute another mediating pathway. Migrants’ participation in local sports activities may lead to improved income levels and socioeconomic status, helping them obtain more stable resource security and broader social opportunities, thereby improving their living conditions and social participation, and creating more favorable external conditions for social integration (34, 35).

However, relatively little attention has been directed toward the subjective feelings and psychological fluctuations experienced by migrant populations during the adaptation process. Some studies, focusing on self-efficacy, identify psychological capabilities as a crucial mechanism. These studies suggest that participation in sports can enhance self-efficacy by fostering social interaction and experiences of control, which in turn improves subjective evaluations such as life satisfaction among internal migrants (36, 37). Nevertheless, fewer studies elucidate how migrants’ willingness to engage socially and their level of integration are shaped by psychological mechanisms, including well-being and satisfaction. Consequently, this study introduces subjective well-being and life satisfaction as mediating variables.

Subjective well-being (SWB) refers to an individual's overall experience of happiness regarding their life circumstances, typically comprising higher levels of positive affect, lower levels of negative affect, and a global evaluation of life (38). SWB is not static but is influenced by factors such as daily experiences, social support, the quality of environmental interactions, and behavioral participation, exhibiting dynamic changes across different life situations (39). Physical activity is considered an important behavioral pathway to enhance SWB. Regular physical exercise can lead to more stable feelings of pleasure and happiness by increasing psychological energy levels, enhancing emotional regulation, and buffering negative emotional experiences (40). The consistency and regularity of physical activity participation are significantly correlated with higher levels of SWB; sustained participation is more likely to accumulate positive experiences and promote increased well-being (41). Furthermore, indirect forms of participation, such as sports spectatorship, can also enhance an individual's well-being through shared group atmosphere and collective emotions, bringing pleasure, excitement, and psychological satisfaction (42).

Further research suggests that subjective well-being (SWB) serves not only as a crucial indicator of individual mental health but also as a significant factor influencing social interaction tendencies and the process of social integration. Individuals exhibiting higher levels of well-being are generally more inclined to engage in social interactions and cultivate stable relationships, which facilitates the development of social trust and a sense of belonging, thereby enhancing their social integration (43, 44). In contrast, those with lower levels of well-being are more prone to social withdrawal and limited interaction, which diminishes their opportunities to access social networks and obtain social support, ultimately impeding their social integration (45). Consequently, this study posits Hypothesis 2:

**H2:** Subjective well-being mediates the relationship between sports participation (including physical exercise and sports spectatorship) and social integration.

### 2.4 Hypothesis 3

Life satisfaction, defined as an individual's subjective assessment of their current life, serves as a crucial mediating variable in elucidating the effects of sports participation on social integration. It encapsulates migrants’ perceptions and evaluations of their quality of life and overall circumstances in their new environment, subsequently influencing their willingness to engage socially, their behaviors related to social participation, and their confidence in the integration process (46, 47). Engaging in physical exercise can enhance individuals’ positive evaluations regarding life order, quality of life, and future prospects by improving their physical and mental well-being, as well as their daily functional performance, thereby augmenting their overall life satisfaction. When individuals develop more stable psychological expectations concerning their life circumstances, they are more inclined to actively participate in social settings and demonstrate a greater willingness to foster ongoing interactions and relationships within communities and public spaces (48, 49).

Sports spectatorship, while an indirect form of sports participation, does not directly yield exercise benefits; instead, it creates an interactive environment that promotes shared attention and experiences, thereby fostering social connections and mutual recognition among spectators. Relationships that arise from shared interests often evolve into more stable emotional bonds and a collective identity, which in turn broadens opportunities for social interaction and enhances individuals’ positive evaluations of their living conditions in the destination (31, 50). Research indicates that individuals’ favorable assessments of their living conditions are significantly correlated with higher levels of social participation and integration. Furthermore, more stable social connections and interactive participation facilitate the development of a lasting sense of belonging and identity within the destination (51, 52).

In the context of internal migration, migrants must navigate rapid adjustments to their lifestyles and social norms, while also confronting the potential inadequacy of support stemming from weakened relationship networks. At this juncture, individuals’ subjective assessments of their living conditions are likely to significantly influence their decisions regarding social interactions and participation, thereby affecting the quality of their social integration (53). Furthermore, related research suggests that as the urban-rural development gap gradually narrows, the explanatory power of economic factors concerning the social integration of the internal migrant population diminishes, whereas the significance of social factors, including social relationships and psychological perceptions, becomes more pronounced (54, 55). Accordingly, this study posits Hypothesis 3:

**H3:** Life satisfaction mediates the relationship between sports participation (physical exercise and sports spectatorship) and social integration.

### 2.5 Hypothesis 4

Physical activity is widely recognized as a daily behavior that contributes positively to both mental and physical well-being. Engaging in regular exercise can alleviate stress and diminish adverse psychological responses, such as anxiety and depression. Furthermore, it enhances physical function and energy levels, facilitating individuals’ ability to maintain a stable and positive mental state (56, 57). Enhanced physical and mental well-being can yield immediate psychological relief, improve an individual's capacity to navigate life challenges and adapt socially in new environments, and establish a more robust psychological and behavioral foundation for social integration (58).

Sports participation typically arises from an individual's voluntary will and active engagement, resulting in spontaneous and non-coercive interactions that distinguish these activities from daily tasks driven by external obligations. Consequently, sports participants are more likely to engage in communication, collaboration, and interaction during activities, which broadens their social networks, fosters interpersonal trust, and cultivates stable, positive experiences that enhance subjective well-being through ongoing joint participation (59–61). The continuous accumulation of positive experiences enables individuals to develop favorable feelings and psychological energy reserves regarding their living conditions, allowing them to approach daily life with a more optimistic attitude and behavioral inclination. This shift further improves their satisfaction with the quality of life and overall circumstances in their environment (62–64).

Increased life satisfaction indicates that individuals’ assessments of their current living conditions are more stable, fostering sustained confidence in their ability to adapt to the environment of their destination. Furthermore, individuals are more likely to engage in interactions, accumulate social connections, and participate in community and public activities, thereby facilitating relationship building and identity formation during the process of social integration (65, 66). Accordingly, this study proposes Hypothesis 4:

**H4:** Subjective well-being and life satisfaction play a chain mediating role between sports participation (physical exercise and sports spectatorship) and social integration.

## 3 Research design

### 3.1 Data source

The data for this study were sourced from the 2023 Chinese General Social Survey (CGSS 2023). The CGSS is a nationwide, continuous, and comprehensive academic survey project organized by the China Survey and Data Center at Renmin University of China. It is designed to systematically collect multi-level information on society, communities, families, and individuals, and to monitor changes in social structure and quality of life in urban and rural China (67). For this analysis, only the subsample of the internal migrant population was selected. Utilizing the identification approach established in prior research, this study employed relevant questions from the CGSS regarding “place of household registration/local residence” to identify the internal migrant population. Specifically, respondents whose registered residence (hukou) was not in the city (district/county) of their current residence were classified as migrants (68). After excluding samples with missing key variables, a final valid sample of 1,660 respondents was achieved, comprising 545 males (32.8%) and 1,115 females (67.2%), with a mean age of 57.47 years (SD = 15.947).

### 3.2 Variable measurement

The dependent variable in this study is “social integration.” In developing this variable, we thoroughly examined the multidimensional aspects of social integration, the essential measurement components of acculturation models, and the operational feasibility of the CGSS data. Following Entzinger and Biezeveld's (69) four-dimensional integration framework, social integration was categorized into four dimensions: “economic foundation,” “political participation,” “interpersonal communication,” and “identity recognition.” This operationalization also referred to Yang's (70) study on the social integration of China's floating population, which measured social integration through multidimensional indicators. In this study, the specific CGSS items included “your current socioeconomic status,” “whether you voted in the last neighborhood committee/village committee election,” “the frequency of social and recreational activities with neighbors,” “the frequency of social and recreational activities with other friends,” and “the social status of the people with whom respondents frequently associated.” The mean score across these four dimensions was utilized to represent the overall level of social integration, with higher scores reflecting a greater degree of integration. The specific items and their corresponding assignments are detailed in Table 1.

**Table 1:** Variable definitions and measurements.

Variable	Measurement items	Assignment instructions
Social integration (economic foundation)	Your current socioeconomic status	Lower layer = 1; Lower-middle layer = 2; Middle layer = 3; Upper-middle layer = 4; Upper layer = 5
Social integration (political participation)	Did you vote in the last neighborhood committee/village committee election?	No = 0; Yes = 3
Social integration (interpersonal communication)	How often do you engage in social and recreational activities with your neighbors?	Never = 0; Once a year or less = 1; Several times a year = 2; About once a month = 3; Several times a month = 4; Once or twice a week = 5; Almost every day = 6
	How often do you engage in social and recreational activities with other friends?	(Same as above)
Social integration (identity recognition)	Apart from relatives, how would you describe the social status of the people you frequently associate with?	Most of these people have a higher social status than you = 1; Most of these people have the same social status as you = 3; Most of these people have a lower social status than you = 5
Physical exercise	In the past year, how often did you engage in the following leisure activity: participating in physical exercise?	Never = 0; Several times a year or less = 1; Several times a month = 3; Several times a week = 6; Daily = 9
Sports spectatorship (live)	In the past year, how often did you engage in the following leisure activity: watching live sports games?	Never = 0; Several times a year or less = 1; Several times a month = 3; Several times a week = 6; Daily = 9
Subjective well-being	Overall, do you feel happy with your life?	Very unhappy = 1; Somewhat unhappy = 2; Not sure if happy or unhappy = 3; Somewhat happy = 4; Very happy = 5
Life satisfaction	I feel very comfortable and at ease now; there aren't many things in my life that cause me worry.	Strongly disagree = 1; Somewhat disagree = 2; Somewhat agree = 3; Strongly agree = 4

The independent variable in this study is “sports participation.” Building on prior research, this investigation differentiates between direct and indirect participation, which correspond to individuals’ physical exercise behaviors and sports spectatorship behaviors, respectively (68, 71). The specific CGSS items included “participating in physical exercise” and “watching live sports games” during the past year. The specific items and assignments are presented in Table 1, with higher scores indicating a greater frequency of participation. The mediating variables are “subjective well-being” and “life satisfaction.” Subjective well-being was measured using the CGSS item “Overall, do you feel happy with your life?”, referring to prior CGSS-based research that used the happiness item to measure subjective well-being (72). Life satisfaction was measured using the CGSS item “I feel very comfortable and at ease now; there are not many things in my life that cause me worry.” The measurement of life satisfaction referred to prior CGSS-based research on sports participation and life satisfaction among internal migrants (71). Detailed descriptions of the specific items and assignments can be found in Table 1, where higher scores reflect elevated levels of subjective well-being and life satisfaction.

## 4 Statistical analysis

All variables in this study were derived from self-reported data collected through a single questionnaire, which may be susceptible to common method bias influenced by factors such as consistency, motivation, and social expectations. Consequently, before conducting data analysis, Harman's one-factor test and the variance inflation factor (VIF) test were employed to evaluate the extent of common method bias. Descriptive statistics and Pearson correlation analyses were executed using IBM SPSS Statistics 23.0.

To test the research hypotheses, mediation effects were analyzed using the PROCESS macro for SPSS. A serial mediation model (Model 6) was employed to evaluate the sequential mediating paths (Preacher & Hayes, 2008). During the estimation process, the confidence interval (CI) was set at 95%, and the bootstrap method with 5,000 resamples was utilized to obtain robust estimates of the indirect effects. In the model specification, social integration served as the dependent variable, while sport participation was entered separately as physical exercise and live sport spectatorship. Subjective well-being and life satisfaction functioned as mediating variables, with gender and age included as control variables. A mediation effect was deemed significant if the 95% bootstrap confidence interval did not encompass zero (73). Furthermore, considering that the mean age of the respondents was 57.47 years (SD = 15.947), the sample included both working-age and older adult populations. Significant differences may exist across age groups regarding social participation patterns and access to resources. Following the conventional threshold used in Chinese population statistics, which classifies individuals aged 60 and above as elderly, this study adopted 60 years as the cutoff for age-based heterogeneity analysis.

## 5 Results

### 5.1 Common method bias test

Harman's one-factor test revealed that the variance accounted for by the first common factor in the unrotated principal component analysis was 30.847%, which is below the commonly accepted threshold of 40%. This finding suggests the absence of severe common method bias in this study (74). Additionally, the variance inflation factor (VIF) was employed to evaluate the risk of multicollinearity. The results indicated that VIF values for all variables ranged from 1.034 to 1.141, with a maximum VIF of 1.141, significantly lower than the criterion of 5. This outcome suggests that the model did not experience substantial multicollinearity and that the estimated relationships among variables were relatively robust (75).

### 5.2 Correlation analysis of sports participation, subjective well-being, life satisfaction, and social integration

Pearson correlation analyses were performed using SPSS Statistics 23.0, with the results summarized in Table 2. The findings indicated a significant positive correlation between physical exercise and live sports spectatorship (*r* = 0.178, *p* < 0.01). Additionally, physical exercise demonstrated significant positive correlations with subjective well-being, life satisfaction, and social integration (*r* = 0.090, *p* < 0.01; *r* = 0.105, *p* < 0.01; *r* = 0.128, *p* < 0.01, respectively). Furthermore, live sports spectatorship exhibited a significant positive correlation with social integration (*r* = 0.064, *p* < 0.01), although its correlations with subjective well-being and life satisfaction were not significant (*r* = 0.048, *p* > 0.05; *r* = 0.044, *p* > 0.05). Meanwhile, subjective well-being was significantly positively correlated with life satisfaction (*r* = 0.343, *p* < 0.01) and social integration (*r* = 0.162, *p* < 0.01); life satisfaction also showed a significant positive correlation with social integration (*r* = 0.126, *p* < 0.01).

**Table 2:** Descriptive statistics and correlation matrix.

variable	M	SD	1	2	3	4	5
Physical exercise	3.47	3.65	1				
Live sports spectatorship	0.45	1.20	0.178**	1			
Subjective well-being	3.94	0.81	0.090**	0.048	1		
Life satisfaction	2.82	0.86	0.105**	0.044	0.343**	1	
Social integration	2.54	0.83	0.128**	0.064**	0.162**	0.126**	1

** *P* < 0.01.

SD, Standard deviation.

### 5.3 The relationship between sports participation and social integration: testing the chain mediation effect

**Figure 1:**
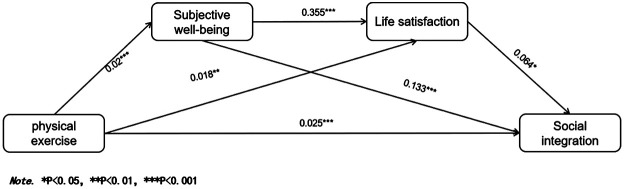
Serial mediation model of subjective well-being and life satisfaction in the relationship between physical exercise and social integration.

**Figure 2:**
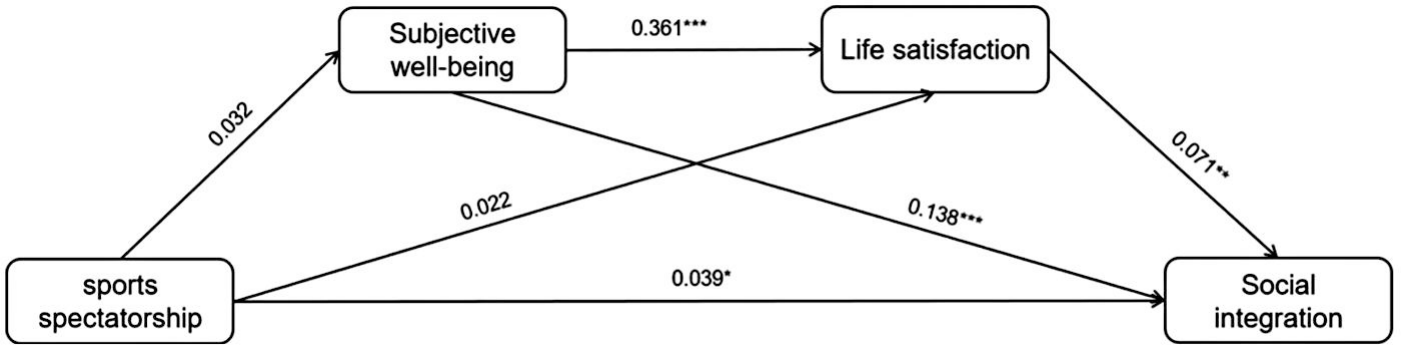
Serial mediation model of subjective well-being and life satisfaction in the relationship between live sport spectatorship and social integration.

A serial mediation model was utilized to investigate the mechanisms by which subjective well-being and life satisfaction affect the relationship between sport participation and social integration. The regression results are detailed in Table 3, and the two tested models are shown in Figure 1 and Figure 2, respectively. The results of the bootstrap mediation effect test are provided in Table 4.

**Table 3:** Regression analysis results.

Dependent variable	Independent variable	R	R2	*F*	Effect		95% CI	
					*B*	*T*	LLCI	ULCI
Subjective well-being	Physical exercise	0.092	0.008	4.696	0.020***	3.635	0.009	0.031
	Watching sports events live	0.0520	0.003	1.519	0.032	1.920	−0.001	0.065
Life satisfaction	Physical exercise	0.358	0.128	60.780	0.018**	3.306	0.007	0.029
	Subjective well-being				0.355***	14.640	0.308	0.403
	Watching sports events live	0.351	0.123	58.166	0.022	1.324	−0.011	0.054
	Subjective well-being				0.361***	14.873	0.313	0.408
Social integration	Physical exercise	0.214	0.046	15.912	0.025***	4.554	0.014	0.036
	Subjective well-being				0.133***	5.093	0.082	0.184
	Life satisfaction				0.064*	2.572	0.015	0.113
	Watching sports events live	0.193	0.037	12.730	0.039*	2.317	0.006	0.072
	Subjective well-being				0.138*	5.253	0.086	0.189
	Life satisfaction				0.071**	2.862	0.022	0.120

* *P* < 0.05.

** *P* < 0.01.

*** *P* < 0.001.

**Table 4:** Bootstrap analysis of mediation effects.

Path	Effect	BootSE	95% CI lower	95% CI upper
Mediation Effect 1 (Physical Exercise)	0.00426	0.00100	0.00200	0.00700
Physical exercise → SWB → Social integration	0.00265	0.00100	0.00100	0.00500
Physical exercise → SWB → LS → Social integration	0.00045	0.00023	0.00007	0.00097
Physical exercise → LS → Social integration	0.00150	0.00100	0.00000	0.00200
Mediation Effect 2 (Live Spectatorship)	0.00700	0.00300	0.00100	0.01400
Live spectatorship → SWB → Social integration	0.00400	0.00300	−0.00100	0.01000
Live spectatorship → SWB → LS→Social integration	0.00200	0.00100	−0.00100	0.00500
Live spectatorship → LS → Social integration	0.001	0.001	−0.002	0.004

SWB, subjective well-being; LS, life satisfaction.

The regression analysis revealed that physical exercise significantly and positively predicted subjective well-being [*B* = 0.020, *t* = 3.635, 95% CI (0.009, 0.031), *p* < 0.001] and life satisfaction [*B* = 0.018, *t* = 3.306, 95% CI (0.007, 0.029), *p* < 0.01]. Furthermore, subjective well-being significantly predicted life satisfaction [*B* = 0.355, *t* = 14.640, 95% CI (0.308, 0.403), *p* < 0.001]. In the social integration model, the direct effect of physical exercise was also significant [*B* = 0.025, *t* = 4.554, 95% CI (0.014, 0.036), *p* < 0.001]. Additionally, both subjective well-being [*B* = 0.133, *t* = 5.093, 95% CI (0.082, 0.184), *p* < 0.001] and life satisfaction [*B* = 0.064, *t* = 2.572, 95% CI (0.015, 0.113), *p* < 0.05] significantly and positively predicted social integration. The serial mediation model for physical exercise is shown in Figure 1.

Bootstrap results indicated that the total indirect effect of physical exercise on social integration was significant [effect = 0.00426, 95% CI (0.002, 0.007)]. When examining specific pathways, the indirect effect of “physical exercise → subjective well-being → social integration” was significant [effect = 0.00265, 95% CI (0.001, 0.005)], representing 62.21% of the total indirect effect. Similarly, the indirect effect of “physical exercise → life satisfaction → social integration” was significant [effect = 0.00150, 95% CI (0.000, 0.002)], accounting for 35.21% of the total indirect effect. Additionally, the serial indirect effect of “physical exercise → subjective well-being → life satisfaction → social integration” was significant [effect = 0.00045, 95% CI (0.00007, 0.00097)], constituting 10.56% of the total indirect effect. These findings suggest that physical exercise fosters social integration through a sequential mechanism involving “enhanced well-being → improved satisfaction.”

In the context of live sports spectatorship, regression analyses indicated that it did not significantly predict subjective well-being [*B* = 0.032, *t* = 1.920, 95% CI (−0.001, 0.065)] or life satisfaction [*B* = 0.022, *t* = 1.324, 95% CI (−0.011, 0.054)]. Conversely, its direct effect on social integration was significant [*B* = 0.039, *t* = 2.317, 95% CI (0.006, 0.072), *p* < 0.05]. Furthermore, both subjective well-being [*B* = 0.138, *t* = 5.253, 95% CI (0.086, 0.189), *p* < 0.001] and life satisfaction [*B* = 0.071, *t* = 2.862, 95% CI (0.022, 0.120), *p* < 0.01] were found to significantly and positively predict social integration. The serial mediation model for live sport spectatorship is shown in Figure 2.

The bootstrap test revealed that the total indirect effect of live sports spectatorship on social integration was significant [effect = 0.007, 95% CI (0.001, 0.014)], constituting 15.22% of the overall effect. However, this significance appeared to arise from the aggregation of several weak pathways, as none of the specific indirect pathways achieved statistical significance: “live spectatorship → subjective well-being → social integration” [effect = 0.004, 95% CI (−0.001, 0.010)], “live spectatorship → life satisfaction → social integration” [effect = 0.001, 95% CI (−0.002, 0.004)], and “live spectatorship → subjective well-being → life satisfaction → social integration” [effect = 0.002, 95% CI (−0.001, 0.005)]. These findings indicate that the mediating mechanism of live sports spectatorship lacks robust support, suggesting that its influence on social integration is predominantly direct.

### 5.4 Heterogeneity analysis of different age groups

To assess the robustness of the findings across various age groups, the sample was categorized into an older adult group (≥60 years) and a non-older adult group (<60 years). Bootstrap analyses were performed utilizing PROCESS Model 6, which involved 5,000 resamples and a 95% confidence interval. The results are detailed in Tables 5, 6.

**Table 5:** Bootstrap analysis of mediation effects in the older adult group (≥60 years).

Path	Effect	BootSE	95% CI lower	95% CI upper
Mediation Effect 1 (Physical Exercise)	0.00394	0.001712	0.001091	0.007792
Physical exercise → SWB → Social integration	0.001761	0.001203	0.000024	0.004662
Physical exercise → SWB → LS → Social integration	0.000488	0.000362	−0.000035	0.001339
Physical exercise → LS → Social integration	0.001691	0.001077	−0.000046	0.00409
Mediation Effect 2 (Live Spectatorship)	0.009514	0.004569	0.001588	0.019261
Live spectatorship → SWB → Social integration	0.005826	0.003651	0.0003	0.014508
Live spectatorship → SWB → LS → Social integration	0.00193	0.001314	0.000071	0.005135
Live spectatorship → LS → Social integration	0.001758	0.002442	−0.00329	0.006853

SWB, subjective well-being; LS, life satisfaction.

**Table 6:** Bootstrap analysis of mediation effect test in non- older group (<60 years).

Path	Effect	BootSE	95% CI lower	95% CI upper
Mediation Effect 1 (Physical Exercise)	0.004805	0.001738	0.001657	0.008497
Physical exercise → SWB → Social integration	0.003687	0.001468	0.00109	0.006872
Physical exercise → SWB → LS → Social integration	0.000425	0.000323	0.000064	0.001191
Physical exercise → LS → Social integration	0.000694	0.000659	−0.000291	0.002282
Mediation Effect 2 (Live Spectatorship)	0.003742	0.005259	−0.006742	0.013845
Live spectatorship → SWB → Social integration	0.00229	0.004445	−0.006675	0.011183
Live spectatorship → SWB → LS → Social integration	0.000272	0.00062	−0.000884	0.001678
Live spectatorship → LS → Social integration	0.00118	0.001757	−0.001834	0.005355

SWB, subjective well-being; LS, life satisfaction.

The results indicated that physical exercise exerted a significant total indirect effect in both older and non-older adult groups [older group: effect = 0.00394, 95% CI [0.00109, 0.00779]; non-older group: effect = 0.00481, 95% CI [0.00166, 0.00850]]. Concerning specific pathways, both groups significantly supported the mediating pathway of “physical exercise → subjective well-being → social integration” [older group: effect = 0.00176, 95% CI [0.00002, 0.00466]; non-older group: effect = 0.00369, 95% CI [0.00109, 0.00687]]. The pathway “physical exercise → life satisfaction → social integration” was not significant in either group. The serial pathway “physical exercise → subjective well-being → life satisfaction → social integration” was significant in the non-older group but not in the older group, indicating that the serial mechanism was less stable across age groups than the pathway mediated by subjective well-being alone.

In the context of live sports spectatorship, the total indirect effect was significant for the older adult group [effect = 0.00951, 95% CI (0.00159, 0.01926)]. Both the pathway “live spectatorship → subjective well-being → social integration” and the serial pathway “live spectatorship → subjective well-being → life satisfaction → social integration” achieved statistical significance. Conversely, the total indirect effect was not significant for the non-older adult group [effect = 0.00374, 95% CI (−0.00674, 0.01385)], and none of the three specific indirect pathways received statistical support.

Overall, the analyses by age group suggest that the primary psychological mechanism through which physical exercise affects social integration is mediated by subjective well-being, a mechanism that remains relatively stable across different age groups. In contrast, the indirect effect of live sports spectatorship was statistically significant only in the older adult group.

## 6 Discussion

### 6.1 Key findings and theoretical explanation

The social integration of migrants is shaped by various factors. This study demonstrates that participation in sports significantly enhances the social integration of China's internal migrant population, with a more pronounced effect observed for physical exercise. Specifically, increased frequency of physical exercise is correlated with elevated levels of social integration among internal migrants. This finding is consistent with prior research (76). Additional studies have indicated that physical exercise positively affects the individual development of diverse target groups, categorized by occupation, gender, and age (77, 78), and these positive effects exhibit a notable degree of stability. Such evidence supports the implementation of sports-related interventions designed to improve social integration within the domestic migrant population. Moreover, the results indicate that the direct effects of both physical exercise and live sports spectatorship on social integration are statistically significant, suggesting that various forms of sports participation provide viable pathways to enhance social integration. Thus, Hypothesis 1 is supported.

Subjective well-being and life satisfaction serve as serial mediators in the relationship between sports participation and the social integration of internal migrants, thereby confirming Hypothesis 4. While the detrimental effects of inadequate social integration on migrants’ mental health and social adaptation have garnered significant attention (66), internal migrants continue to encounter integration challenges. These challenges include weak social connections, insufficient community participation, and limited identity recognition, which arise from institutional constraints and disparities in resource accessibility (53, 79). Migrants are not merely passive recipients of external environmental influences; their increased initiative significantly impacts adaptation outcomes and the quality of integration (80, 81). Participation in sports can serve as a constructive choice, offering migrant populations relatively stable public interaction spaces. Through repeated engagement, sports participation fosters the generation and maintenance of social networks, thereby facilitating emotional support (82, 83). This involvement enhances subjective well-being by increasing socio-emotional support and cultivating a sense of belonging (84). Elevated levels of subjective well-being contribute to more favorable cognitive evaluations of one's life circumstances, which in turn results in greater life satisfaction (9, 85). Increased life satisfaction may also diminish sensitivity and defensiveness toward social exclusion (9), thereby enhancing willingness and confidence in social participation and ultimately promoting higher levels of social integration.

The results of the heterogeneity test indicate that the indirect effect of “physical exercise → subjective well-being → social integration” is significant in both older and non-older adult groups. This finding suggests that subjective well-being maintains a relatively stable explanatory power in the relationship between physical exercise and social integration. In contrast, although the serial mediation path “physical exercise → subjective well-being → life satisfaction → social integration” was significant in the full sample, neither this serial path nor the path mediated solely by life satisfaction received statistical support in the age-grouped samples. The psychological mechanism through which physical exercise influences social integration demonstrates a degree of convergence following age stratification: the mediating role of subjective well-being appears more stable, whereas pathways involving life satisfaction may be more vulnerable to variations in sample composition and individual heterogeneity.

### 6.2 Differential mechanisms for different forms of sports participation

While both physical exercise and attendance at sporting events represent significant forms of sports participation, the latter is more limited by time and financial resources. Opportunities for individuals to attend sporting events are closely linked to their socioeconomic status (86). Our findings reveal that attendance at sporting events exerts a significant direct positive influence on the social integration of internal migrants; however, the pathways through which it indirectly affects subjective well-being and life satisfaction lack support, indicating that Hypotheses 2 and 3 are only partially substantiated. It is essential to recognize that attending events may incur costs such as ticket prices, transportation, and opportunity costs, which could restrict the frequency of participation among the migrant population and subsequently undermine the stability of its psychological mechanisms.

The age-group analyses conducted in this study revealed heterogeneity in the mechanisms underlying live sports spectatorship. The indirect effects of live spectatorship exhibited asymmetry across the grouped samples, achieving statistical significance solely within the older adult cohort. Specifically, in this older group, the indirect effect of “live spectatorship → subjective well-being → social integration” was significant, and the serial indirect effect of “live spectatorship → subjective well-being → life satisfaction → social integration” was also supported. In contrast, the non-older group did not demonstrate statistically significant total, direct, or related indirect effects of live spectatorship. These findings suggest that the psychological transmission mechanism linking live spectatorship to social integration may be more contingent upon individuals’ age and social context.

The results of the mediation effect test further reveal that the mechanisms through which physical exercise and live sports spectatorship influence the social integration of internal migrants are divergent. Physical exercise not only directly enhances integration levels but may also bolster migrants’ motivation for integration and the quality of their experiences through the accumulation of positive encounters and improved assessments of their living conditions. According to embodied cognition theory, an individual's physical state significantly affects their cognitive processes (87). Regular physical exercise improves cardiopulmonary function, strengthens muscle strength, and promotes the development of motor and sensory systems (88). Furthermore, under specific conditions of intensity, frequency, and duration, it increases individual vitality and enhances positive emotional experiences (89). This sustained positive feeling, resulting from an improved physical state, facilitates migrants’ access to emotional support in daily interactions, thereby providing ongoing psychological motivation and a behavioral foundation for their social integration.

In contrast, although live sports spectatorship significantly influences social integration, its predictive capacity for subjective well-being and life satisfaction is not substantial, and related indirect and serial mediation effects remain unsupported. While attending live sports events can elicit positive emotional responses such as excitement, passion, and exhilaration, these feelings are primarily driven by the event's atmosphere and the collective experience. Ramchandani et al. (9) noted that the positive experiences associated with live sports viewing are characterized by their strong contextuality and brief duration. Observing sports events does not equate to the physical engagement, skill acquisition, and challenge-overcoming processes inherent in active participation. Since spectators do not engage directly in teamwork, endurance maintenance, or stress coping, their viewing experiences are less likely to serve as stable psychological resources or emotional support. Consequently, this diminishes the potential for significant enhancement of subjective well-being and its subsequent translation into sustained improvements in social integration. When considering these findings alongside age-group analyses, it can be further inferred that the psychological benefits of live sports viewing may more readily translate into increased subjective well-being and subsequently influence social integration among older adults, whereas younger adults are less likely to establish a stable mechanism for such effects.

These findings resonate with previous research on sport and migrant integration. Spaaij's (8) study of Somali Australians showed that sport is more likely to contribute to integration when it helps migrants develop social capital beyond existing ethnic and friendship-based networks. Studies of Swiss sport clubs also suggest that sport settings are not automatically integrative; rather, their social effects depend on membership experiences, interaction opportunities, organizational openness, and the broader club climate (10, 11). Corvino et al. (12) further indicated that sport participation may support immigrants’ sense of community and well-being when it creates inclusive and meaningful social interactions. Compared with these studies, the present findings further suggest that sport participation should not be treated as a homogeneous behavior. Physical exercise may form a more stable psychological pathway because it is more easily embedded in everyday routines, neighborhood spaces, and repeated interpersonal encounters. Live sport spectatorship, by contrast, may generate shared emotions, social identity, and perceived social support, but these benefits are often tied to particular events, teams, venues, and collective viewing contexts (30, 31, 42, 90). Therefore, live spectatorship may directly promote social integration, but it may not readily accumulate into stable psychological resources unless it is embedded in repeated community interaction. In this sense, the findings add nuance to sport–migration research by showing that physical exercise and spectator participation may contribute to migrant integration through different social and psychological routes.

### 6.3 Theoretical contributions and practical implications

This study makes several significant contributions to the literature. First, it differentiates between physical exercise and live sports spectatorship, integrating the latter into the explanatory framework of social integration for migrant populations. It also examines the positive impact of sports participation on social integration, thereby broadening the research perspective on how such participation facilitates the social adaptation of migrant groups and enhancing the understanding of variations in forms of sports engagement. Second, this research deepens the comprehension of the mechanisms through which sports participation influences social integration. While existing studies have highlighted the health benefits and mood-enhancing effects of sports participation, noting that regular exercise can mitigate stress and improve positive emotional states (91, 92), this study clarifies the psychological pathways through which sports participation impacts social integration. It begins with the serial mechanisms of subjective well-being and life satisfaction, thereby further elucidating the social functions of sports.

The findings of this study present practical implications for enhancing the social integration of internal migrants. First, sports participation should not be regarded merely as a means to promote physical health; its diverse benefits in fostering positive emotional experiences, enhancing emotional support and social well-being, and cultivating a sense of belonging must also be highlighted. Second, the government should enhance the availability of public sports facilities and community sports services, reduce participation barriers through public welfare and inclusive sports activities, and further expand community-level avenues for exercise and spectator engagement. These measures would enable the migrant population to attain a greater sense of well-being and a more stable sense of belonging (12, 93). According to the sample results presented in this paper, the overall level of sports participation among internal migrants is relatively low, particularly regarding insufficient attendance at live spectator events, which may significantly limit the impact of spectator participation on subjective well-being and life satisfaction. Third, professional social organizations and community entities should enhance the organization of sports activities and provide scientific exercise guidance. This approach would ensure continuous and accessible sports support for the migrant population, mitigate the risk of sports injuries and over-fatigue, and thereby improve the safety and sustainability of sports participation, making it a more reliable resource for promoting social integration (94).

### 6.4 Limitations and future research directions

This study has certain limitations that should be addressed in future research. First, this study uses cross-sectional data from the CGSS 2023; therefore, caution is required when making causal inferences about the relationships among variables. Future research should consider integrating longitudinal tracking data or employing experimental and quasi-experimental designs, along with more rigorous identification strategies, to enhance the causal explanatory power of the findings. Additionally, key variables such as sports participation, subjective well-being, life satisfaction, and social integration are derived from single-item self-reported measurements. These measures may inadequately capture the complexities of these concepts and are susceptible to social desirability bias and recall error, thereby introducing the risk of common method bias (74). Future studies could incorporate multi-source data collection methods, including community activity records, sports platform data, or objective measures of spectator behavior, to enhance measurement reliability and interpretative robustness (73). Furthermore, this paper's assessment of sports participation primarily focuses on participation frequency, neglecting to differentiate among intensity, duration, and type of physical exercise, as well as failing to explore varying effects across different spectator contexts. This limitation constrains the precise identification of which forms of sports participation are most beneficial for social integration. Future research should incorporate intensity and contextual dimensions alongside frequency, while also comparing the effects of various participation types on subjective well-being, life satisfaction, and social integration. This approach would yield more targeted evidence for effective interventions aimed at enhancing social integration among the migrant population through sports participation. Furthermore, the conclusions of this paper are derived from the overall migrant population within the CGSS sample. Subsequent studies must assess the applicability and robustness of these findings among young migrant populations or migrant groups with varying income levels to enhance the generalizability and policy relevance of the conclusions.

### 6.5 Conclusion

This study, utilizing data from the CGSS 2023, investigated the influence of sports participation on the social integration of China's internal migrant population. The findings indicated that both physical exercise and attendance at sporting events significantly facilitated social integration; however, the impact of physical exercise proved to be more consistent. Mechanism analysis further indicated that physical exercise enhances social integration through the mediating effects of subjective well-being and life satisfaction, with subjective well-being exhibiting greater explanatory power across various age groups. Attendance at sporting events primarily demonstrated a direct effect within the overall sample, although its psychological transmission pathway lacked consistent support; nonetheless, some evidence of indirect effects was identified within the older adult group. These results imply that sports participation, particularly regular daily exercise, may serve as a crucial behavioral pathway for enhancing the social integration of the migrant population.

## Data Availability

The data that support the findings of this study are available from the corresponding author Dr. Wu upon reasonable request. The data that support the findings of this study are available from the Chinese General Social Survey. Restrictions apply to the availability of these data, which were used under license for this study. Data are available from the authors with the permission of Chinese General Social Survey.
